# Carotid Artery Stenting: Evolution, Evidence, and Contemporary Practice in the Era of Intensive Medical Therapy

**DOI:** 10.3390/life16040601

**Published:** 2026-04-04

**Authors:** Sakshi Dixit, FNU Anamika, Anmol Multani, Akiva Rosenzveig, Bargavi Kathirvel, Suprita Degala, Manvitha Thalamati, Lee Kirksey, Christopher Bajzer, Daniel Raskin, Aravinda Nanjundappa

**Affiliations:** 1 Cleveland Clinic Akron General, Akron, OH 44307, USA; 2 Department of Cardiology, Cleveland Clinic, Cleveland, OH 44195, USAsupritadegala27@gmail.com (S.D.); muntalamati@gmail.com (M.T.); bajzerc@ccf.org (C.B.); 3 Department of Radiology, Cleveland Clinic, Cleveland, OH 44195, USA

**Keywords:** carotid artery stenting, transcarotid artery revascularization, carotid endarterectomy

## Abstract

Carotid artery stenosis remains a major cause of ischemic stroke worldwide, and its management continues to evolve in parallel with advances in surgical, endovascular, and medical therapies. Carotid endarterectomy (CEA) was established as the standard of care for symptomatic high-grade stenosis following landmark randomized trials, while carotid artery stenting (CAS) subsequently emerged as a less invasive alternative for appropriately selected patients. This review aims to summarize the historical evolution of carotid artery stenting, critically appraise evidence from major clinical trials comparing CAS and CEA, and examine contemporary practice patterns in the era of intensive medical therapy. A comprehensive review of randomized trials, registries, guideline statements, and recent literature was performed to synthesize current evidence regarding procedural outcomes, patient selection, and emerging technologies, including transcarotid artery revascularization (TCAR). Large, randomized trials have demonstrated comparable long-term composite outcomes between CAS and CEA in selected patients, although peri-procedural risk profiles differ, with higher stroke risk observed after CAS and higher myocardial infarction rates after CEA. Technological advancements in embolic protection devices, stent platforms, and alternative access strategies have further refined endovascular approaches. Concurrently, improvements in intensive medical therapy—including lipid-lowering, antiplatelet therapy, blood pressure control, smoking cessation, and lifestyle modification—have substantially reduced overall stroke risk, particularly in asymptomatic patients. In the contemporary era, optimal stroke prevention requires individualized, multidisciplinary decision-making that integrates symptom status, anatomical complexity, comorbid conditions, procedural expertise, and sustained long-term vascular risk factor management following revascularization.

## 1. Introduction

Atherosclerosis is the most common pathological process affecting the carotid arteries. It is a major cause of carotid artery stenosis, with an estimated global prevalence of approximately 21% among individuals aged 30–79 years [1]. Atherosclerotic carotid artery disease is a major contributor to stroke both in the United States and worldwide, with prevalence increasing with advancing age and occurring more frequently among men, as well as White and Native American populations [2]. According to the Society of Vascular Surgery, major risk factors for carotid atherosclerosis include hypertension, diabetes mellitus, cigarette smoking, hyperlipidemia, and increasing age. Male sex and family history of stroke are also significant risk factors [3]. In a large population-based cohort, Luedemann et al. demonstrated that unfavorable lifestyle patterns, including physical inactivity and poor diet, were independently associated with increased asymptomatic carotid atherosclerosis (OR 2.68), highlighting the substantial contribution of behavioral risk factors to carotid disease burden [4]. Evidence further suggests that dietary patterns rich in fruits, vegetables, and fish are generally associated with lower carotid intima-media thickness (CIMT), whereas diets high in processed foods and refined grains trend toward increased CIMT, highlighting the potential influence of overall diet on early carotid atherosclerosis, though evidence remains heterogeneous and largely non-significant [5]. The American Society of Radiology classifies carotid artery stenosis into mild (<50% occlusion), moderate (50–69% occlusion), and severe (>70% occlusion) based on duplex carotid Doppler findings [6]. Although carotid artery stenosis may remain asymptomatic in a subset of patients, it can lead to devastating neurologic complications in others [7]. Cerebrovascular events may occur due to embolization from unstable atherosclerotic plaques, resulting in transient monocular blindness (amaurosis fugax), transient ischemic attack (TIA), or ischemic stroke, or through hemodynamic compromise from high-grade stenosis, causing cerebral hypoperfusion and watershed infarction [7,8,9]. Collectively, extracranial carotid artery disease accounts for approximately 10–20% of all ischemic strokes [9]. Management of carotid stenosis involves an interplay between medical, endovascular, and surgical management. Historically, carotid endarterectomy (CEA) was considered the gold standard for both symptomatic and selected asymptomatic patients based on robust evidence from landmark trials, including the North American Symptomatic Carotid Endarterectomy Trial (NASCET), European Carotid Surgery Trial (ECST), Asymptomatic Carotid Atherosclerosis Study (ACAS), and Asymptomatic Carotid Surgery Trial (ACST) [3,10].

Carotid artery stenting (CAS) has subsequently emerged as a minimally invasive alternative, particularly attractive for patients considered high risk for surgical intervention, to treat atherosclerotic carotid disease and reduce stroke risk [11]. The procedure involves advancing a catheter to the carotid artery, dilating the stenotic lesion with balloon angioplasty, and deploying a stent to maintain vessel patency [7,11]. Over the past three decades, CAS has evolved substantially through advances in device design, embolic protection strategies, and peri-procedural medical management protocols, positioning CAS as an important alternative to CEA in appropriately selected patients [12].

Improvements in embolic protection systems, stent platforms, and the development of transcarotid artery revascularization (TCAR) have refined endovascular approaches and expanded therapeutic options. Concurrently, intensive medical therapy—including high-intensity lipid lowering, optimized antiplatelet regimens, blood pressure control, and aggressive risk factor modification—has significantly reduced stroke risk, particularly among asymptomatic individuals.

Despite these advances, important limitations persist within the evidence base. Many landmark randomized trials were conducted in an era preceding contemporary intensive medical therapy and did not systematically stratify or adjust for behavioral cardiovascular risk factors such as dietary patterns, sedentary behavior, tobacco use, alcohol consumption, sleep disorders, and psychosocial stress. These modifiable factors are strongly associated with carotid atherosclerosis progression and plaque vulnerability. Their underrepresentation in trial design and randomization frameworks introduces potential residual confounding and may limit the generalizability of results to modern populations receiving comprehensive preventive care. Furthermore, comorbidity burden is often reported descriptively without detailed integration of behavioral risk modification strategies into outcome interpretation.

In the modern era, where stroke prevention is increasingly driven by aggressive vascular risk factor control, the relative benefit of procedural revascularization must be contextualized within optimized medical therapy and long-term lifestyle intervention. This creates a critical knowledge gap: how should clinicians interpret historical trial data when applied to contemporary patients receiving substantially improved preventive care? Additionally, the evolving role of CAS, particularly with newer technologies and patient selection strategies, requires re-evaluation against this backdrop of declining baseline stroke risk.

The novelty of this review lies in its integration of procedural evidence with contemporary advances in intensive medical therapy and behavioral risk modification. Rather than examining CEA and CAS in isolation, we synthesize historical and modern data to reassess their roles within current stroke prevention paradigms.

We hypothesize that in the era of optimized medical therapy and comprehensive risk factor modification, patient selection—incorporating symptom status, anatomical characteristics, procedural risk, and behavioral risk profiles—has become more critical than the choice of revascularization modality alone.

Therefore, the objective of this review is to (1) trace the evolution of carotid artery stenting, (2) critically appraise comparative evidence between CAS and CEA, (3) examine technological and procedural advancements including TCAR, (4) highlight limitations of landmark trials in the context of modern preventive strategies, and (5) define the contemporary role of carotid revascularization within an individualized, multidisciplinary stroke prevention framework.

## 2. Historical Perspectives

The development of CAS represents a progression from experimental technique to established clinical practice. As operator experience with balloon angioplasty and intravascular stent placement expanded, attention turned toward the carotid arteries as a potential target for percutaneous revascularization, particularly in patients considered poor surgical candidates. The first carotid balloon angioplasty procedures were performed in the early 1980s [13,14]. However, it was soon recognized that angioplasty alone was insufficient due to higher rates of complications, including restenosis, vessel wall recoil, angiographically evident intimal dissection, and plaque dislodgement with particulate embolization [15]. In parallel lines with coronary stents, intravascular scaffolds were proposed to stabilise intravascular plaque, prevent collapse, and maintain patency of the vessel. The Carotid and Vertebral Artery Transluminal Angioplasty Study (CAVATAS) demonstrated a higher incidence of restenosis associated with angioplasty alone as compared to angioplasty combined with stent placement [16]. The first balloon-expandable stent was deployed in the carotid artery in 1989. Early reports suggested that these stents were prone to extrinsic compression; major adverse events, defined as death, stroke, or myocardial infarction within 30 days of the procedure, occurred in more than 10% of patients at 30 days [17,18,19]. By 1994, CAS was determined to be technically feasible, but the incidence of neurological sequelae was a serious problem [18]. These complications were attributed to procedural factors, including stenosis of the passage due to the catheter, shortening of self-expanding stents after deployment, and compression caused by struts of the stents against the atheromatous plaque during balloon dilatation, which might dislodge plaque debris or cause local thrombus [10]. Collectively, these early experiences underscored the need for specialized devices and refined procedural techniques tailored to the unique anatomical and hemodynamic characteristics of the carotid circulation.

In 2004, the Stenting and Angioplasty with Protection in Patients at High Risk for Endarterectomy (SAPPHIRE) trial compared the outcomes of CAS vs. CEA in high-risk patients [12]. A total of 334 high-risk patients with either symptomatic > 50% stenosis or asymptomatic with >80% stenosis were randomly assigned to CAS vs. CEA groups. The results from the trial demonstrated that CAS, performed along with specific emboli protection devices, was non-inferior to CEA for high-risk patients [15]. Based on these findings, the U.S. Food and Drug Administration (FDA) approved the use of CAS on 31 August 2004, for patients with ≥50% symptomatic or ≥80% asymptomatic carotid stenosis who were considered high risk for CEA due to anatomical or anatomic or medical risks [20,21]. Subsequently, on 17 March 2005, Center of Medicare and Medicaid Services (CMS) also approved the coverage CAS for symptomatic patients with 70% or greater stenosis who were considered high risk for CEA in the opinion of a surgeon. These two steps were the defining moment in adoption for CAS as an alternative to CEA in certain patient populations [21].

The Stent-Protected Angioplasty vs. Carotid Endarterectomy (SPACE) trial was a prospective, randomized controlled trial that aimed to determine whether CAS was non-inferior to CEA in patients with severe (≥70%) symptomatic carotid artery stenosis [22]. The trial sought to evaluate whether a less invasive endovascular approach like CAS could achieve comparable outcomes to CEA. It was conducted across 35 centers in three countries (Germany, Switzerland, and Austria) and enrolled 1200 patients. Although it was originally planned to enroll 2500 participants, the enrollment was discontinued early due to funding limitations, reducing the statistical power required to confirm non-inferiority [23,24]. The primary endpoint was ipsilateral ischemic stroke or death from any cause within 30 days of treatment. The secondary endpoints were perioperative adverse events and the two-year risk of restenosis, stroke, and death. The primary endpoint event rate was 6.34% in the CEA group and 6.84% in the CAS group (*p* = 0.09 for non-inferiority). Across multiple assessed endpoints, CEA showed more favorable outcomes than CAS [23]. Also, the perioperative stroke or death rates in both groups exceeded the accepted 6% complication threshold for symptomatic carotid revascularization, raising concerns regarding procedural safety within the study context [23]. The SPACE trial thus failed to demonstrate the non-inferiority of CAS compared with CEA in patients with symptomatic high-grade carotid stenosis. While the short-term outcomes were numerically comparable, statistical significance of equivalence was not achieved.

There are a few limitations that may have influenced the results. The low rate of usage of cerebral embolic protection devices (27%) could have potentially contributed to the higher complication rates [23,24]. Additionally, early termination due to lack of funding limited the statistical power of the trial [23,24]. A substantially larger sample size would have been required to definitively establish non-inferiority and validate the results [24].

Overall, although the SPACE trial was one of the largest independently funded randomized studies in this field at the time, its methodological constraints and its limitations reduce the strength of its conclusions.

The Carotid Revascularization Endarterectomy vs. Stenting Trial (CREST) was a large, multicenter, randomized controlled trial that compared CAS and CEA in patients with carotid artery stenosis [25]. The study was conducted across 108 centers in the United States and 9 in Canada. A total of 2500 patients were followed. It aimed to determine whether CAS was comparable to CEA in preventing major vascular events in both symptomatic and asymptomatic patients. CREST initially enrolled patients with symptomatic carotid stenosis. Due to slow recruitment, eligibility was expanded to include asymptomatic patients with higher stenosis thresholds. By the end of enrollment, approximately 47% of participants in each group were asymptomatic [23,26].

The primary composite endpoint was periprocedural stroke, myocardial infarction (MI), or death, plus ipsilateral stroke within 4 years. The estimated 4-year rates of the primary endpoint were 7.2% in the CAS group and 6.8% in the CEA group (*p* = 0.51), demonstrating no statistically significant difference between the two interventions. However, in the periprocedural period, CAS was associated with a higher risk of stroke, whereas CEA was associated with a higher risk of MI.

Overall, CREST demonstrated no significant difference between CAS and CEA in the composite outcomes, supporting CAS as an alternative to CEA in selected patients. These findings led to revisions of U.S. guidelines in 2011, recommending CAS as a reasonable alternative to CEA in selected patients at a low surgical risk [23].

However, several methodological concerns have been raised regarding the CREST trial: (i) The mid-trial inclusion of asymptomatic patients diluted the original cohort. Due to the differing natural histories and treatment benefits between symptomatic and asymptomatic stenosis, this may have reduced the statistical power to detect meaningful differences in higher-risk subgroups [23,26]. (ii) The inclusion of myocardial infarction as a primary endpoint and equating it with stroke, despite stroke’s greater impact on quality of life, has also been criticized [23,26]. Moreover, the purpose of CAS and CEA is to prevent strokes (as well as death resulting from strokes), not MI [26]. (iii) Subsequent subgroup analyses demonstrated higher stroke and death rates with CAS among symptomatic patients, women, and patients aged ≥65 years. When these groups are excluded, comparable outcomes were primarily observed in asymptomatic men under 65 years of age, a population in whom medical therapy alone may be sufficient [23,26]. (iv) CAS patients received substantially higher doses of dual antiplatelet therapy than CEA patients, which may partially explain the lower perioperative MI rates in the CAS group [26].

Also, the CAS technology employed in CREST, including the stents and embolic protection approaches, is now largely considered outdated. With advancements such as improved embolic protection devices, newer stent designs, and better patient selection, CAS outcomes are expected to improve [26]. Nevertheless, additional trials are required to confirm this potential and to establish the equivalence or superiority of CAS relative to CEA.

CREST-2 consisted of two parallel, multicenter, observer-blinded randomized trials conducted across 155 centers in five countries [27]. The study aimed to determine whether adding carotid revascularization (CAS or CEA) to intensive medical management reduces the risk of stroke compared with intensive medical management alone in patients with high-grade (≥70%) asymptomatic carotid stenosis. Eligible participants had high-grade (≥70%) asymptomatic carotid stenosis without recent stroke or transient ischemic symptoms. The primary endpoints were perioperative stroke or death and ipsilateral stroke within 4 years. The results demonstrated that, among asymptomatic patients, CAS combined with intensive medical therapy reduced the risk of the composite outcome compared with medical therapy alone. In contrast, CEA did not demonstrate a statistically significant benefit over intensive medical therapy alone.

In the era of aggressive risk-factor control, the results of CREST-2 support the role of carotid artery stenting, when performed with appropriate operator expertise and anatomical selection, as a valid revascularization strategy in asymptomatic high-grade carotid stenosis. However, the findings do not imply that all asymptomatic patients require intervention, nor that stenting should universally replace endarterectomy [28]. CREST-2 emphasises individualized, shared decision-making, integrating modern intensive medical therapy with selective revascularization in appropriately chosen asymptomatic patients [28].

Collectively, these landmark trials established the foundation for carotid revascularisation strategies and helped shape the current guideline recommendations (Table 1).

## 3. Present Day Practice: Medical Optimization and Stenting

In a large cohort of patients with asymptomatic severe carotid artery stenosis treated with modern medical therapy, the yearly risk of ipsilateral ischemic stroke was approximately 0.9%, with a 5-year cumulative risk of 4.7% (2.7% after accounting for competing risks), demonstrating a significant reduction in stroke risk achievable with medical optimization alone [29].

More recently, the CREST-2 trial demonstrated that intensive medical therapy alone results in low stroke risk in patients with asymptomatic high-grade carotid stenosis, and that the addition of carotid artery stenting further decreased the 4-year incidence of ipsilateral stroke or death (6.0% vs. 2.8%), emphasizing that optimized medical therapy remains the foundation of management prior to procedural intervention [27].

According to current ESC and AHA/ASA guidelines, intensive medical optimization in carotid artery stenosis involves antiplatelet therapy, high-intensity statins, strict blood pressure and glycemic control, and lifestyle interventions such as diet, exercise, and smoking cessation.

Lifestyle modification, a core component of intensive medical optimization in carotid artery stenosis, is complementary to pharmacologic therapy. Current guidelines recommend adherence to a heart-healthy dietary pattern (Mediterranean-style diet rich in fruits, vegetables, whole grains, and unsaturated fats), regular aerobic physical activity (≥150 min per week of moderate-intensity exercise), smoking cessation, and weight management as part of the best medical therapy [30,31]. In a large cohort of adults aged ≥55 years with vascular risk factors, a healthy diet was associated with a 14% relative reduction in stroke risk over a median follow-up of approximately 5 years, independent of statin and antiplatelet use, underscoring the additive benefit of dietary modification [30]. Smoking cessation and regular physical activity are associated with reductions in overall vascular events and are now considered an essential part of modern best medical therapy protocols for asymptomatic carotid artery stenosis management [31,32,33]. These studies collectively support the inclusion of lifestyle interventions as a standard component of medical optimization aimed at reducing long-term stroke risk in carotid stenosis.

Hypertension is an independent risk factor for carotid artery stenosis [34]. The 2025 American Heart Association/American College of Cardiology (AHA/ACC) Hypertension Guideline recommends a target blood pressure of <130/80 mm Hg in patients with cardiovascular disease to reduce the risk of stroke and other vascular events [35]. In patients with symptomatic carotid stenosis, higher blood pressure was associated with increased recurrent stroke risk, suggesting benefit from blood pressure reduction; however, in those with severe bilateral carotid stenosis, lower blood pressure was paradoxically associated with increased stroke risk, likely reflecting impaired cerebral perfusion [36]. Notably, patients with carotid artery stenosis have also been shown to have a high prevalence of renovascular hypertension and coexisting renal artery stenosis [37]. This is relevant as renin–angiotensin system inhibitors, such as ACE inhibitors and ARBs, used for lowering blood pressure, effectively treat renovascular hypertension as well. Per the HOPE trial, daily ramipril (10 mg) reduced the risk of cardiovascular death, myocardial infarction, or stroke by ∼22% compared with placebo in high-risk patients without heart failure, with significant reductions in each component and in all-cause mortality over ~5 years [38]. Additionally, in the LIFE trial, losartan significantly reduced the risk of cardiovascular death, stroke, and myocardial infarction in hypertensive patients with left ventricular hypertrophy, with the greatest benefit observed in lowering stroke risk [39].

Statin therapy is a cornerstone of intensive medical management in carotid artery stenosis, with established roles in both primary and secondary prevention of ischemic stroke. In SPARCL, investigators found high-dose atorvastatin (80 mg daily) reduced recurrent stroke by 16% and major cardiovascular events by 20% in patients with prior ischemic stroke or transient ischemic attack, supporting aggressive lipid lowering for secondary cerebrovascular prevention [40]. In asymptomatic carotid stenosis, evidence from various studies demonstrates that statin-based pharmacotherapy is associated with increased plaque stability, reduced plaque progression, and ipsilateral stroke rates compared with historical controls, supporting its role in primary stroke prevention [31]. Reflecting these data, current American Heart Association/American Stroke Association and Society for Vascular Surgery guidelines recommend high-intensity statin therapy for all patients with carotid stenosis irrespective of symptom status, with treatment goals of ≥50% reduction in LDL-cholesterol and an absolute LDL-C target < 70 mg/dL, while European Society of Cardiology/European Atherosclerosis Society guidelines advocate a more aggressive LDL-C target of <55 mg/dL in very-high-risk patients, including those with significant carotid stenosis. When LDL targets are not achieved with statins alone, adjunctive lipid-lowering therapy with ezetimibe or PCSK9 inhibitors is recommended by both US and European guidelines [30,31].

A systematic overview by the Antiplatelet Trialists’ Collaboration showed that, in patients treated with antiplatelet therapy following a transient ischemic attack or ischemic stroke, there was a 17% relative reduction in the risk of major cardiovascular events, defined as myocardial infarction, stroke, or vascular death [41]. However, evidence for aspirin use in asymptomatic carotid artery stenosis is weak. In the asymptomatic Cervical Bruit Study, 372 neurologically asymptomatic patients with ≥50% carotid stenosis on duplex ultrasound were randomized to receive either enteric-coated aspirin (325 mg/day, *n* = 188) or placebo (*n* = 184) and followed for a median of 2.3 years. There were no significant differences between groups in the annual rate of all ischemic events and death (11.0% with aspirin vs. 12.3% with placebo; *p* = 0.61), in rates of vascular events alone (10.7% vs. 11.0%; *p* = 0.99), or in adjusted hazard ratios for the composite end point (HR 0.99; 95% CI, 0.67–1.46; *p* = 0.95). These findings indicate that aspirin did not confer a protective effect against clinical ischemic events in this asymptomatic high-grade carotid stenosis cohort [42]. Conversely, in the Asymptomatic Carotid Emboli Study (ACES), among 477 patients with asymptomatic carotid stenosis followed for 2 years, antiplatelet therapy and lower mean blood pressure were independently associated with a reduced risk of ipsilateral stroke and TIA, with antiplatelet use lowering events by approximately 50% compared with untreated patients [43]. Despite these mixed results, aspirin is the most widely used antiplatelet agent in asymptomatic carotid stenosis [44].

Short-term dual antiplatelet therapy with aspirin combined with clopidogrel or ticagrelor has shown to reduce the risk of recurrent ischemic strokes in patients with acute noncardioembolic transient ischemic attack or ischemic stroke [45,46]. However, long-duration dual antiplatelet therapy is associated with an unfavorable risk-to-benefit ratio in patients with symptomatic cerebrovascular disease [47,48]. Consequently, the primary role of P2Y12 receptor antagonists in asymptomatic carotid stenosis is limited to patients who are allergic or intolerant to aspirin. Given the uncertain balance of benefits and risks of antiplatelet therapy in ACS, one approach is to offer it selectively to patients with high-risk plaques identified on imaging [31].

According to the 2021 American Diabetes Association guidelines, most adults with type 2 diabetes who are not at high risk for hypoglycemia should aim for a hemoglobin A1c of less than 7.0% [49]. Tight glycemic control (HbA1c < 7%) reduces microvascular complications, though effects on macrovascular outcomes are less certain. Hypoglycemia increases cardiovascular risk, so it should be minimized, especially in high-risk patients. Glucose-lowering therapy should prioritize agents with proven cardiovascular benefit (e.g., GLP-1 receptor agonists, SGLT2 inhibitors) and low hypoglycemia risk, with individualized targets based on life expectancy and overall risk [50].

Carotid artery stenting is a minimally invasive percutaneous endovascular procedure performed under fluoroscopic guidance to treat hemodynamically significant carotid artery stenosis and reduce the risk of ischemic stroke. Vascular access is typically obtained via the common femoral artery, and a guide catheter is advanced into the common carotid artery. Following diagnostic angiography, an embolic protection device, most commonly a distal filter, is deployed beyond the stenotic lesion to reduce the risk of cerebral embolization. The lesion is then crossed with a guidewire and may undergo balloon pre-dilation prior to deployment of a self-expanding stent across the stenosis, with post-dilation if needed to optimize luminal expansion. Final angiography confirms adequate stent apposition, restoration of flow, and absence of distal embolization. Intraprocedural hemodynamic monitoring is essential to minimize complications such as hypotension, bradycardia, and embolic stroke [51].

Traditionally, the transfemoral approach has been the preferred method for carotid artery stenting, demonstrating acceptable success rates [52]. A systematic review and meta-analysis including 6917 patients demonstrated no significant differences between transradial and transfemoral carotid artery stenting with respect to peri-procedural stroke (1.7% vs. 1.9%; odds ratio 0.98, 95% confidence interval 0.49–1.96), mortality (1.0% vs. 0.9%; odds ratio 0.95, 95% confidence interval 0.38–2.37), myocardial infarction (0.2% vs. 0.3%; odds ratio 1.53, 95% confidence interval 0.20–11.61), transient ischemic attack (0.4% vs. 1.0%; odds ratio 0.46, 95% confidence interval 0.11–1.95), or access-site complications (2.2% vs. 1.0%; odds ratio 0.97, 95% confidence interval 0.48–1.98), supporting the overall safety and efficacy of the transradial approach [53]. Building on these findings, expert consensus highlights that the technical advantages of transradial carotid artery stenting are most pronounced in patients with unfavorable aortic arch anatomy, difficult transfemoral access such as severe aortoiliac occlusive disease or morbid obesity, and in those at increased bleeding risk. However, the radial approach is associated with more radiation exposure, underscoring that radial access may be considered a complementary strategy rather than a substitute for transfemoral access, requiring individualized procedural planning [12].

The optimal duration of dual antiplatelet therapy following carotid artery stenting was assessed by Fukutome et al. in 2024 [54] by directly assessing neointimal coverage of stents using angioscopy. In a prospective cohort of 20 patients, angioscopic evaluation at 2 months post-stenting revealed complete neointimal coverage in 18 of 19 evaluable cases, while noninvasive imaging such as ultrasonography and angiography detected neointima less reliably. No peri-procedural or post-stenting complications were observed. These findings suggest that a 2-month course of dual antiplatelet therapy may generally be sufficient to achieve protective neointimal coverage on carotid stents, although the authors note that only a single dual-layer stent type was studied and recommend further research across various stent designs [54].

In a nationwide cohort study of 12,034 patients undergoing carotid artery stenting, Yoo et al. found that the duration of dual antiplatelet therapy (aspirin plus clopidogrel) after the procedure did not significantly affect the composite rate of ischemic stroke, gastrointestinal bleeding, or intracranial hemorrhage within 12 months. Specifically, the primary outcome occurred in 2.5% of patients receiving short-duration therapy (3 months to <6 months) vs. 2.1% of those on long-duration therapy (≥6 months) (adjusted hazard ratio 0.87, 95% confidence interval 0.65–1.16; *p* = 0.337), supporting the use of a shorter course of dual antiplatelet therapy after stenting without increased adverse events [55]

In patients with asymptomatic carotid artery stenosis, a 2022 systematic review and meta-analysis of 7 randomized trials (7230 patients: 3920 CAS vs. 3198 CEA) found that the overall peri-operative composite of stroke, myocardial infarction, and death was similar between CAS and CEA (OR 1.13; 95% CI 0.87–1.47; *p* = 0.37). CAS was associated with a higher risk of any peri-procedural stroke (OR 1.62; 95% CI 1.16–2.24; *p* = 0.004) and nondisabling stroke (OR 1.81; 95% CI 1.23–2.65; *p* = 0.003), while there were no significant differences in disabling stroke, death, or long-term composite outcomes. These findings suggest that CAS is a less invasive alternative to CEA with broadly comparable overall safety and efficacy, but carries a higher short-term stroke risk that requires careful patient selection and procedural expertise [56].

Transcarotid artery revascularization (TCAR) has emerged as a hybrid surgical–endovascular technique designed to address several limitations associated with transfemoral carotid artery stenting (CAS) [57]. The procedure involves direct surgical exposure of the common carotid artery through a small longitudinal or transverse incision above the clavicle, followed by the introduction of a specialized sheath into the artery and, most often, the femoral vein in the groin (sometimes the internal jugular vein). These two sheaths are connected to a flow-control device with a filter, and the patient is given intravenous heparin to prevent clotting. The key safety step is “flow reversal,” during which the carotid artery is temporarily clamped above the puncture site so blood flows away from the brain, through the filter, and back into the vein, which helps capture any debris. With flow reversal running, the operator crosses the narrowing, deploys the stent, and may perform balloon angioplasty. This FDA-cleared system (ENROUTE Transcarotid Neuroprotection System, cleared in 2015) reduces the need to pass catheters through the aortic arch and, together with flow reversal before crossing the lesion, may lower the risk of embolic stroke compared with transfemoral carotid stenting [58].

**Figure 1:**
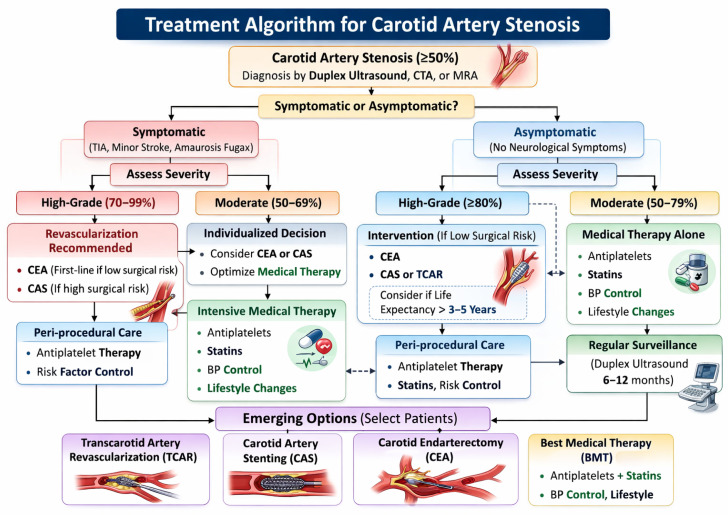
Treatment Algorithm for Carotid Artery Stentosis.

Based on the symptom status, degree of stenosis, surgical risk, and anatomic consideration, a structured approach to management can help guide appropriate revascularisation strategy selection (Figure 1).

TCAR is favored over the carotid endarterectomy (CEA) due to a favorable peri-procedural safety profile, as it minimizes cerebral embolization. Traditional transfemoral CAS requires catheter navigation through the aortic arch and supra-aortic vessels that can dislodge atherosclerotic debris, particularly in elderly patients or those with complex arch anatomy. TCAR avoids arch manipulation entirely, significantly reducing procedural embolic burden. Flow reversal provides continuous cerebral protection during lesion crossing, angioplasty, and stent deployment, which are often the procedures traditionally associated with the highest embolic risk [59].

Despite these promising findings, TCAR was not evaluated in earlier randomized trials, such as CREST and CREST-2, which shaped modern carotid revascularization guidelines. As a result, long-term durability, restenosis rates, and outcomes in standard-risk and asymptomatic patients remain areas of active investigation. Ongoing studies are increasingly focused on defining optimal patient selection criteria and determining whether TCAR should be expanded beyond high-risk cohorts into broader clinical practice [60].

Parallel to advances in access strategies, significant innovation has occurred in carotid stent design. As early as the 1980s, carotid percutaneous intervention was performed using the Early balloon angioplasty, which was later replaced by balloon-expandable stents and early self-expanding platforms such as the Wallstent. Balloon-expandable stents were soon phased out due to their susceptibility to external compression, limited conformability at bifurcations, and increased risk of vessel injury, which was replaced by self-expanding stents, which offer superior adaptability to carotid anatomy and resistance to extrinsic forces. These stents undergo major metamorphosis over the decades, evolving into the modern self-expanding carotid stents, constructed from nitinol, and available in open-cell, closed-cell, and hybrid configurations. Open-cell stents provide excellent flexibility and vessel wall conformability, making them suitable for tortuous anatomy and angled bifurcations; however, they have reduced plaque scaffolding. Closed-cell stents offer superior plaque coverage and predictable radial force, which is advantageous for soft or ulcerated plaques, but they are relatively stiff. Hybrid stents combine these properties by incorporating a closed-cell mid-segment for plaque stabilization with open-cell proximal and distal segments to enhance flexibility and vessel adaptation. Clinical experience and prospective studies have shown that the lesion-specific device selection based on individual lesion morphology, plaque composition, and supra-aortic anatomy reduces peri-procedural and early post-procedural neurological events. In particular, hybrid stents have shown favorable safety profiles as the Multicenter registry data reported high technical and procedural success rates with low 30-day stroke and death rates in high-risk populations, including elderly patients, those with ulcerated plaques, and patients with tortuous carotid anatomy [61,62]. Newer-generation carotid stents incorporate dual-layer or micromesh designs intended to address these shortcomings. These stents consist of an external scaffold for radial strength and vessel conformity combined with an inner micromesh layer that functions as an embolic containment system. The reduced free-cell area limits plaque extrusion and may decrease the incidence of both clinical and subclinical embolization [63]. Procedural enhancements extend beyond stent architecture. Advances in embolic protection devices (EPDs), including proximal occlusion systems and flow reversal technologies, aim to provide more comprehensive cerebral protection than distal filter-based systems alone [64]. Alternative access routes, such as radial artery access for diagnostic angiography and selective interventions, are under investigation to further reduce access-site complications and improve patient comfort [65].

Optimal selection between carotid endarterectomy (CEA), transfemoral carotid artery stenting (CAS), and transcarotid artery revascularization (TCAR) is dependent on patient-specific factors. Data from CREST and subsequent analyses suggest that patients aged ≥70 years have a higher peri-procedural stroke risk with transfemoral CAS due to increased aortic arch atherosclerosis and vascular tortuosity, making CEA or TCAR more preferred in this population [12,20,26]. Conversely, younger patients (<65–70 years) tend to have comparable or more favorable outcomes with CAS [25].

Sex-specific differences have also been observed, with women demonstrating a higher peri-procedural risk with CAS compared to CEA, although the absolute differences are modest [12,26].

Anatomical considerations are important to procedural selection. Patients with unfavorable aortic arch anatomy, severe carotid tortuosity, or extensive calcification are less suitable for transfemoral CAS [10,12,26] and may benefit from TCAR, which avoids aortic arch manipulation and provides cerebral protection via flow reversal [57,58,59]. Similarly, patients with prior neck surgery, radiation therapy, or high carotid bifurcation are often considered high-risk for CEA and may be better candidates for CAS or TCAR [10,12,30].

Collectively, the data supports an individualized approach in which CEA is often favored in older, low-surgical-risk patients; transfemoral CAS in younger patients with favorable anatomy; and TCAR in patients with high surgical risk or challenging aortic arch anatomy.

While duplex ultrasonography is the first-line imaging modality for screening and grading carotid artery stenosis, advanced imaging techniques are increasingly used to refine risk stratification and guide management decisions. Computed tomography angiography (CTA) provides high-resolution visualization of vascular anatomy, plaque calcification, and luminal narrowing, and is useful for procedural planning [6,10]. Magnetic resonance imaging (MRI) enables evaluation of plaque composition by viewing features like lipid-rich necrotic core, intraplaque hemorrhage, and fibrous cap, which indicates plaque vulnerability and increased embolic risk [10].

Integration of these advanced imaging modalities into clinical practice can improve patient selection, procedural planning, allowing for a more personalized approach to carotid intervention [12,30].

The various management strategies for carotid atherosclerosis, including lifestyle modifications, medication therapy, and revascularization approaches have been summarized in Table 2. 

## 4. Discussion

This narrative review synthesizes the historical evolution, clinical evidence, and contemporary management strategies for carotid artery stenosis. Historically, CEA emerged as the gold standard for symptomatic high-grade stenosis following landmark trials such as NASCET and ECST, which demonstrated clear stroke reduction benefits. Subsequent studies, including ACAS and ACST, expanded the role of CEA to selected asymptomatic patients, establishing long-term risk reduction with careful patient selection [7,10,66].

CAS has emerged as a minimally invasive option for the treatment of selected high-risk cases of carotid artery stenosis. Early experiences highlighted significant procedural challenges, including neurological complications, plaque embolization, and stent compression, which prompted the development of dedicated carotid stents and embolic protection devices. Landmark trials such as SAPPHIRE, SPACE, and CREST demonstrated that CAS provides outcomes comparable to CEA in selected patients, while CREST-2 emphasized the importance of intensive medical therapy, refining modern indications for revascularization.

Beyond the procedural interventions, this review also highlights that lifestyle and behavioral risk factors, including smoking, diet, physical activity, and alcohol consumption, significantly influence carotid atherosclerosis progression and clinical outcomes [10,31]. Yet these factors were indeed largely uncontrolled in the landmark CEA trials from the 1990s. This represents a critical gap when applying historical trial data to contemporary practice. A 2025 Chinese cohort study of 43,651 participants demonstrated that adherence to all four healthy lifestyle factors (non-smoking, moderate alcohol, sufficient physical activity, and a healthy diet) reduced carotid plaque incidence by 36% (OR 0.64) and delayed plaque onset [67]. Current guidelines universally recommend Class I evidence-based lifestyle interventions including smoking cessation, Mediterranean diet, 150 min of moderate aerobic activity weekly, and weight reduction as foundational therapy for all patients with carotid stenosis, regardless of whether revascularization is pursued [31,66].

Contemporary practice integrates advanced imaging modalities (duplex ultrasound, CT angiography, and MRI plaque imaging) to identify high-risk plaques and guide intervention strategies. Individualized decision-making, considering symptom status, comorbidity burden, anatomical factors, and procedural risk, is critical to optimizing outcomes. Additionally, Coronary computed tomography angiography (CCTA) allows quantification of atherosclerotic plaque burden and characterization of plaque type, aiding risk stratification for myocardial infarction. Evidence suggests using a CCTA-based staging system may reduce CAD-related morbidity and mortality, despite some knowledge gaps [68]. Similarly, a CCTA-like imaging-based staging system could be developed for carotid artery disease to quantify plaque burden and type, helping to stratify risk of stroke and guide preventive therapies. Novel approaches such as transcarotid artery revascularization (TCAR) further expand options for high-risk patients, although evidence continues to evolve.

In summary, the management of carotid stenosis has progressed from open surgical approaches to a spectrum of minimally invasive interventions supported by intensive medical therapy and lifestyle modification. This review underscores the importance of integrating historical evidence, landmark trial data, and emerging strategies to guide individualized patient care, while highlighting gaps in the literature, particularly regarding behavioral risk factors and preventive strategies. Future research should focus on refining patient selection, advancing device technology, and incorporating lifestyle and imaging-based risk stratification to further reduce the burden of ischemic stroke.

### 4.1. Limitations

Despite providing a comprehensive overview of carotid artery stenting and its contemporary management, several limitations should be noted. As a narrative review, this work does not follow a systematic methodology, and study selection was based on relevance and impact rather than predefined criteria, which may introduce some selection bias.

Many landmark trials comparing CEA, CAS, and TCAR underrepresent key patient groups, including women, older adults, and those with complex aortic anatomy. These studies also largely overlook behavioral and lifestyle factors—such as diet, physical activity, alcohol use, sleep patterns, and emotional health—which could influence outcomes and limit generalizability.

Although we highlight advances in stent technology, embolic protection, and TCAR, the use of advanced imaging in routine practice is still limited. Techniques such as CT angiography (CCTA) or MRI plaque imaging could potentially help assess plaque morphology and vulnerability, but most studies to date focus only on luminal stenosis, leaving gaps in understanding how imaging can improve stroke risk assessment and guide patient selection.

Finally, as with all narrative reviews, our conclusions are descriptive rather than quantitative. Future research combining detailed plaque imaging, longitudinal clinical outcomes, standardized assessment of lifestyle factors, and more diverse patient populations will be essential to improve risk stratification and optimize individualized care.

### 4.2. Future Perspectives

The future of carotid revascularization research is increasingly focused on refining rather than replacing existing therapies. Ongoing and planned trials are evaluating optimized stent platforms, advanced cerebral protection strategies, and patient-centered outcomes, including neurocognitive performance and quality of life.

Large multicenter registries continue to play a critical role in generating real-world evidence, particularly for newer techniques such as TCAR. These data sources complement randomized trials by capturing outcomes across broader patient populations and practice settings. Comparative effectiveness studies are expected to further clarify the relative roles of CEA, transfemoral CAS, and TCAR in specific subgroups, such as elderly patients, women, and those with complex vascular anatomy.

An important area of investigation involves asymptomatic carotid stenosis, where the balance between procedural risk and long-term stroke prevention remains contentious. As medical therapy continues to improve, the threshold for intervention is likely to evolve, emphasizing the need for robust contemporary data.

Future innovations in carotid intervention are expected to focus on improving procedural safety, durability, and reproducibility. Advances in stent biomechanics aim to enhance flexibility, radial force distribution, and fatigue resistance, particularly in tortuous or heavily calcified vessels. Improvements in embolic protection technology, including next-generation flow reversal systems and hybrid protection strategies, may further reduce neurological complications.

Robotic assistance and enhanced imaging guidance represent additional frontiers in carotid intervention. Robotic systems may reduce operator fatigue, improve precision, and limit radiation exposure, while advanced imaging techniques may enable real-time assessment of cerebral perfusion and embolic risk during intervention.

## 5. Conclusions

Carotid artery stenosis remains a significant contributor to ischemic stroke worldwide, emphasizing the importance of risk stratification as well as individualised treatment strategies. Over the past several decades, the management paradigm has shifted from surgical revascularisation options established by landmark trials such as NASCET and ESCT to a multimodal management involving intensive medical optimisation with an evolving role for CAS. Randomized trials including CREST and SPACE demonstrated that CAS offers comparable long-term efficacy to carotid endarterectomy (CEA) in appropriately selected patients, although differences persist in peri-procedural stroke and myocardial infarction risk profiles. With the advent of embolic protection devices, dual-layer and micromesh stents, and the emergence of transcarotid artery revascularization (TCAR), there has been increasing refinement in the safety profile. Nevertheless, optimal patient selection remains paramount. In the contemporary era of aggressive lipid-lowering, antiplatelet therapy, and risk-factor modification, the threshold for intervention continues to evolve.

Ultimately, carotid revascularization should be guided by a patient-centered framework integrating symptom status, anatomical considerations, comorbidity burden, life expectancy, and institutional expertise. Continued randomized investigation and real-world registry data are required to further clarify the relative roles of CEA, transfemoral CAS, and TCAR in modern practice.

Beyond its role in evaluating luminal stenosis, carotid ultrasound has the potential to be used as a tool for early vascular risk assessment and detection of subclinical atherosclerosis. Measurements such as carotid intima-media thickness (CIMT), arterial stiffness, and early plaque formation can identify structural vascular changes that precede clinically overt cardiovascular disease [10,30]. These early alterations reflect cumulative exposure to cardiovascular risk factors and may improve risk stratification beyond traditional clinical scoring systems [30].

In individuals at increased risk, such as those with diabetes, hypertension, or a strong family history of cardiovascular disease, carotid ultrasound can help detect early arterial remodeling before the development of hemodynamically significant stenosis. Prior studies have demonstrated that these subclinical vascular changes are associated with future cardiovascular events and may provide an opportunity for earlier and more aggressive preventive interventions [69,70].

Incorporating carotid ultrasound into preventive cardiovascular assessment frameworks may represent a practical and noninvasive strategy to identify high-risk individuals, guide early medical therapy, and reduce long-term cerebrovascular risk [30,31].

## Figures and Tables

**Table 1:** Landmark Randomized Trials in Carotid Revascularisation.

Trial	Year	Population	Comparison	Primary Endpoint	Key Findings	Clinical Impact
NASCET	1991	Symptomatic ≥ 70% stenosis	CEA vs. medical therapy	Ipsilateral stroke	CEA significantly reduced stroke risk	Established CEA as gold standard for symptomatic high-grade stenosis
ECST	1998	Symptomatic carotid stenosis	CEA vs. medical therapy	Stroke/death	Confirmed benefit of CEA in severe stenosis	Reinforced surgical management
ACAS	1995	Asymptomatic ≥ 60% stenosis	CEA vs. medical therapy	Ipsilateral stroke/death	CEA reduced 5-year stroke risk	Expanded CEA to selected asymptomatic patients
ACST	2004	Asymptomatic stenosis	CEA vs. medical therapy	Stroke risk reduction	Long-term stroke reduction with CEA	Supported surgical intervention in selected asymptomatic patients
SAPPHIRE	2004	High surgical-risk patients	CAS vs. CEA	Death/stroke/MI at 30 days + 1 year	CAS is non-inferior to CEA	Led to FDA approval of CAS in high-risk patients
SPACE	2006	Symptomatic ≥ 70% stenosis	CAS vs. CEA	Stroke/death at 30 days	Failed to prove non-inferiority of CAS	Highlighted need for embolic protection and operator expertise
CREST	2010	Symptomatic + asymptomatic	CAS vs. CEA	Composite: stroke/MI/death	No significant difference overall; CAS ↑ stroke, CEA ↑ MI	Established CAS as alternative in selected patients
CREST-2	Ongoing/Recent	Asymptomatic ≥ 70% stenosis	CAS + medical vs. medical alone; CEA + medical vs. medical alone	Stroke/death	Intensive medical therapy substantially reduces stroke risk; revascularization still beneficial in selected cases	Refined indications for intervention in asymptomatic disease

**Table 2:** Overview of Treatment Strategies for Carotid Atherosclerosis.

Treatment Type	Key Components/Description	Representative Trials/Evidence	Clinical Notes/Outcomes
Lifestyle Modification & Risk Factor Management	Diet, physical activity, smoking cessation, alcohol moderation, blood pressure, diabetes, lipid control	Luedemann et al., 2002 [4]; Bhat et al., 2019 [5]	Improves carotid intima-media thickness (CIMT) and reduces early atherosclerosis; often underrepresented in landmark trials
Medical Therapy	Antiplatelet therapy, statins, blood pressure control, risk factor optimization	CREST-2 (CAS + medical vs. medical alone; CEA + medical vs. medical alone)	Reduces stroke risk, particularly in asymptomatic patients; foundation of modern management
Carotid Endarterectomy (CEA)	Surgical removal of plaque from carotid artery	NASCET 1991; ECST 1998; ACAS 1995; ACST 2004	Gold standard for symptomatic high-grade stenosis; reduces ipsilateral stroke risk; also beneficial in selected asymptomatic patients
Carotid Artery Stenting (CAS)	Percutaneous stent placement with/without embolic protection	SAPPHIRE 2004; SPACE 2006; CREST 2010	Less invasive alternative to CEA; good long-term outcomes in selected patients; peri-procedural stroke risk must be considered
Transcarotid Artery Revascularization (TCAR)	Hybrid approach with direct carotid access and flow reversal	ROADSTER 2015–2019	Emerging technique for high-risk patients; may reduce embolic complications; evidence still evolving

## Data Availability

No new data were created or analyzed in this study. Data sharing is not applicable to this article.
